# Adult Muscle Stem Cells: Exploring the Links Between Systemic and Cellular Metabolism

**DOI:** 10.3389/fcell.2019.00312

**Published:** 2019-12-06

**Authors:** Gunjan Purohit, Jyotsna Dhawan

**Affiliations:** ^1^Centre for Cellular and Molecular Biology, Hyderabad, India; ^2^Institute for Stem Cell Science and Regenerative Medicine, Bengaluru, India

**Keywords:** muscle stem cell, metabolism, oxidative phosphorylation, glycolysis, fatty acid oxidation, autophagy

## Abstract

Emerging evidence suggests that metabolites are important regulators of skeletal muscle stem cell (MuSC) function and fate. While highly proliferative in early life, MuSCs reside in adult skeletal muscle tissue in a quiescent and metabolically depressed state, but are critical for the homeostatic maintenance and regenerative response of the tissue to damage. It is well established that metabolic activity in MuSC changes with their functional activation, but the spatiotemporal links between physiological metabolism and stem cell metabolism require explicit delineation. The quiescent MuSC is defined by a specific metabolic state, which is controlled by intrinsic and extrinsic factors during physiological and pathological tissue dynamics. However, the extent of tissue and organismal level changes driven by alteration in metabolic state of quiescent MuSC is currently not well defined. In addition to their role as biosynthetic precursors and signaling molecules, metabolites are key regulators of epigenetic mechanisms. Emerging evidence points to metabolic control of epigenetic mechanisms in MuSC and their impact on muscle regenerative capacity. In this review, we explore the links between cell-intrinsic, tissue level, and systemic metabolic state in the context of MuSC metabolic state, quiescence, and tissue homeostasis to highlight unanswered questions.

## Introduction

Nutrition is a prerequisite for energy production, homeostasis, and growth. In mammals, prenatal development is largely dependent on the maternal nutritional status, conveyed to the fetus via the placenta. Postnatal growth and overall health status are defined by nutrition received from environmental sources: excess, restricted, or imbalanced nutritional intake negatively impacts a variety of processes, affecting development and adult physiology as well as disease and aging. Nutritional impact on organismal physiology and metabolic flux between highly active tissues such as skeletal muscle and liver are well established. However, there is less evidence for specific links between systemic metabolic status and adult stem cell physiology. Here, we review the evidence for connections between metabolic flux at the level of whole organism and the impact on stem cell chromatin in a manner that controls stem cell contribution to skeletal muscle function and repair.

The importance of skeletal muscle for locomotion, thermoregulation, respiration, and digestion is very well known, but the role of muscle as an important regulator of systemic metabolism is often underappreciated. Alterations in systemic metabolism can generate acute or chronic changes in skeletal muscle (Homsher and Kean, 2003; Mann et al., 2010), and reciprocally, muscle metabolic state can drive changes in systemic metabolism (Zurlo et al., 1990; Homsher and Kean, 2003; Baskin et al., 2015). In humans, liver, skeletal muscle, and brain are the most metabolically active organs, together consuming more than 50% of the available oxygen (Rolfe and Brown, 1997). Skeletal muscle being the largest tissue in most mammals constitutes ∼40% of total body mass and utilizes 20% of available oxygen in the resting state (Field et al., 1939; Melzer, 2011). Skeletal muscle can regulate systemic glucose level by both insulin-dependent or -independent mechanisms and serves as a storage site for glycogen (Defronzo et al., 1981b; Lee et al., 1995). Muscle is also the main reservoir of amino acids and solely fulfills the amino acid requirement of the whole body under conditions of starvation (Ruderman, 1975). Overall, the metabolic activity of skeletal muscle is a function of its contractile frequency, which is controlled by neural activity. To understand the extrinsic regulation of muscle metabolism, a brief description of skeletal muscle tissue components and development is provided below.

Skeletal muscle tissue consists of long myofibers formed by the fusion of muscle precursors (see Box 1 for a brief overview on skeletal muscle development). Thus, in mature muscle fibers, multiple nuclei are embedded in a common cytoplasm that is highly structured and organized into contractile cytoskeletal units known as sarcomeres. Each myofiber is innervated by a single axon, which makes contact with the myofiber at the motor endplate, and through which electrical signals are communicated, leading to a specific frequency of contraction. Associated with myofibers in adult muscle is a rare population of muscle stem cells (MuSCs) also called satellite cells, which are injury-responsive and can robustly repair or replace damaged myofibers.

**BOX 1.**

Development of skeletal muscle and tissue regeneration by muscle stem cells. Embryonic muscle development and adult muscle regeneration by muscle stem cells. Multinucleated myofibers are formed during embryonic development by fusion of mono-nucleated muscle progenitors. In late fetal development, a distinct population of muscle progenitors known as satellite cells or muscle stem cells (MuSC) can be discerned adjacent to muscle fibers, and these progenitors persist postnatal. At birth, MuSC constitute up to 30% of nuclei in the tissue, but this number declines to 1–4% of nuclei as muscle tissue grows during the early postnatal period, by addition of MuSC to fibers via fusion. MuSC distribution can vary depending on species, age, muscle type, and muscle location. In normal adults, muscle tissue is turns over slowly, and this homeostatic replacement is maintained by the small resident MuSC population. After muscle injury, satellite cells get activated by mechanochemical signaling and differentiate into transit-amplifying (TA) cells, which undergo further steps of differentiation and fusion to generate myofibers. During activation, a small population of proliferating myoblasts re-enter into quiescence to maintain MuSC pool. However, under pathological conditions generated during trauma or inherited disease, loss of differentiated muscle fibers necessitates an enhanced response from the resident stem cells as myofibers can only be replaced by expansion of the stem cells followed by their fusion to minimally damaged myofibers or differentiation to replace dying myofibers.

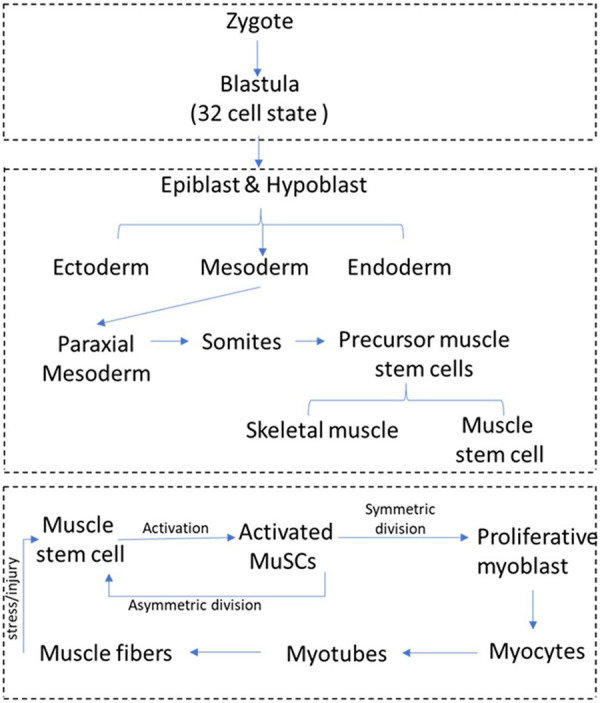

Muscle stem cells are sandwiched between the plasma membrane (sarcolemma) of muscle fibers and the ensheathing basement membrane (Mauro, 1961). In the absence of activation signals such as stress or injury, adult MuSC persist in a quiescent state that is characterized by reversible exit from cell cycle and an overall reduction in cellular activities. Upon activation by damage-induced signals, MuSC emerge from their myofiber niche and start dividing (see Box 1 for a schematic overview of muscle regeneration). MuSC replenish their stem cell pool through asymmetric cell division or divide symmetrically to the larger number of myoblasts required for tissue repair and regeneration (Shinin et al., 2006; Kuang et al., 2007). Proliferating myoblasts travel to the site of muscle injury and differentiate into myocytes, which repair injury either by fusing to damaged myofibers or generate new myofibers by fusing with other myocytes. The surrounding niche and other cell types in muscle such as fibroblasts, macrophages, and endothelial cells play an important role in modulating quiescence and activation of MuSCs (Marazzi and Sassoon, 2012; Yin et al., 2016). Signals from injured muscle are not only perceived by MuSCs in the tissue immediately surrounding the lesion, but may be transferred to uninjured muscles at some distance (as far as the contralateral limb), where they prime MuSC for activation. These primed MuSCs designated “G-alert” to distinguish from the undisturbed “G0” cells respond more rapidly than quiescent MuSCs to muscle (Rodgers et al., 2014). Given that long-range signals from distant sites are capable of modulating MuSC activation, understanding the impact of circulating metabolites on quiescent MuSC is essential.

When considering the metabolic state of muscle tissue, it is important to factor in the requirements and responses of MuSCs as distinct from the differentiated myofibers: although a minor component of the tissue as a whole, they are essential for maintenance of muscle tissue (see Box 2 for a brief overview of major metabolites and metabolic pathways). The low turnover of the rare, resident MuSC population in normal muscle has influenced investigation of their metabolic regulation. Consequently, our understanding of interdependence of skeletal muscle and MuSC metabolism on systemic and niche metabolism is very limited. While there is currently no evidence for this, the possibility remains that under pathological conditions, the metabolic state of MuSCs influence muscle tissue health, which may further impact systemic health.

**BOX 2.**

Major metabolites and metabolic pathways. The three classes of macronutrients (protein, carbohydrates, and fatty acids) are metabolized in cells through different metabolic pathways mainly for the production of energy or for the synthesis of other complex macromolecules. The metabolites generated in one metabolic pathway might be utilized in other pathways. The key metabolites that are generated intracellularly are acetyl-CoA and glycogen, while the key metabolites that are shared via the systemic circulation are free fatty acids, glucose, etc.

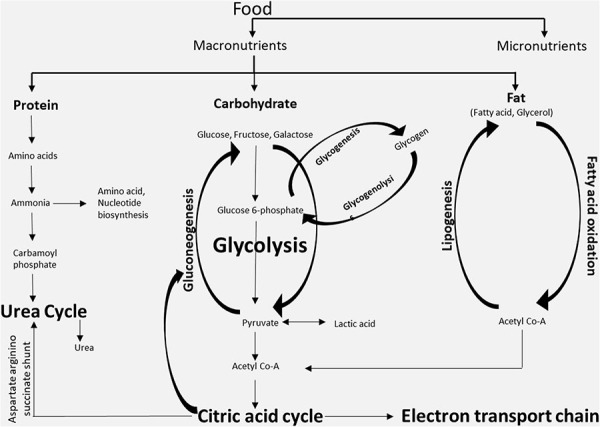

The study of metabolism is no longer limited to the understanding of cellular bioenergetics, but has been extended to metabolite-mediated epigenetic regulation. Epigenetic modifications of chromatin control the accessibility of different regions of the genome to transcriptional and signaling machinery and are a crucial regulatory step in gene expression (Gao et al., 2018; Reid et al., 2018; Schvartzman et al., 2018). A number of metabolites from distinct metabolic pathways can directly participate in DNA or histone modifications as substrates or co-factors (Box 3). Hence, the particular cellular metabolic state at a given point of time and the nutrient-regulated abundance of circulating metabolites constitute another regulatory layer for genome regulation. Therefore, acute or chronic metabolic changes in skeletal muscle would be expected to modulate MuSC gene expression through epigenetic modification, and thereby drive changes in cell fate and function.

**BOX 3.**

Metabolic regulation of epigenetic state. Epigenetic modification includes heritable or dynamic enzymatic modification of DNA or histones. Metabolites play an important role in DNA and histone modification. The product of one-carbon metabolism, S-adenosylmethionine (SAM), donates its methyl group to methylate cytosine in DNA and is thereby converted into S-adenosyl homocysteine (SAH), which reenters the one-carbon cycle. The reverse reaction is catalyzed by DNA methyl transferase (DNMT) where the methyl group of cytosine base is transferred to α-ketoglutarate (a-KG) and converts it into succinate. Both α-KG and succinate are utilized in the TCA cycle. The modification of lysine residue of histone tails modulates DNA binding and protein recruitment. Similar to DNA methylation, SAM donates its methyl group to histones, but that reaction is catalyzed by histone methyltransferases (HMT). Histone demethylases catalyze the reverse reaction to remove methyl groups from histone lysines. The methyl group is received by α-KG in a reaction involving the cofactor FAD. Another important modification of histone protein is the acetylation of lysine residues. Histone acetylation is catalyzed by histone acetyltransferase (HAT), which receives the acetyl group from Acetyl-CoA, an important metabolic product of protein, carbohydrate, or fat metabolism. The deacetylation of histone proteins is catalyzed by histone deacetylase (HDAC) with NAD+ as the cofactor.

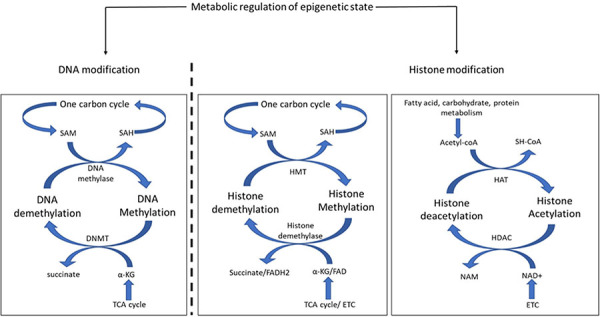

In this review, we assess the available evidence that provides links between systemic, tissue, and stem cell metabolism in the context of skeletal muscle and outline the current understanding of metabolic regulation of MuSC epigenetic state. We also highlight open questions that constitute important avenues of future research with potential translational significance.

## Systemic Metabolism and Skeletal Muscle

The building blocks of skeletal muscle are the myofibers that are bundled into fascicles. Myofibers are long cylindrical multinucleated skeletal muscle cells formed by the differentiation and fusion of precursor myoblasts (Box 1). The process of muscle contraction is a cascade of events initiated by myofiber-associated nerve axons. Since skeletal muscles are connected to bones through tendons, contraction in skeletal muscle leads to movement of the musculoskeletal system. Muscle contraction is a highly energy-intensive process and facilitated by ATP hydrolysis. Circulating free fatty acids and glucose as well as intracellular glycogen and triglycerides serve as the substrates for myofiber ATP production. Therefore, pathological alterations in circulating metabolites such as are seen in diabetes have a major negative impact on skeletal muscle function.

**FIGURE 1:**
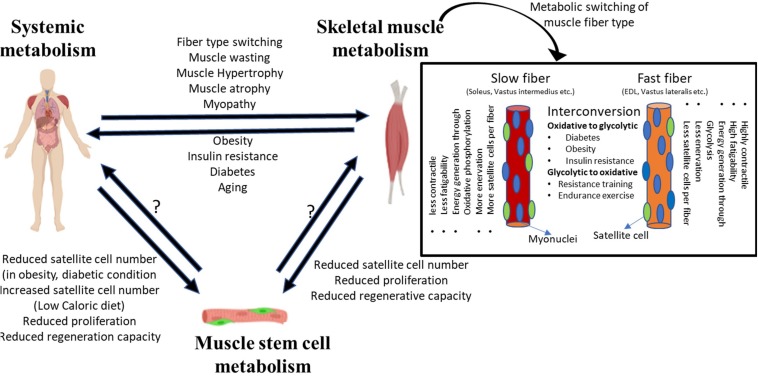
System level metabolism is tightly linked to skeletal muscle metabolism. Alterations in system level metabolism due to stress or pathological conditions can affect muscle physiology. Systemic metabolism can alter the fiber-type composition of muscle, wherein different fiber types depict distinct metabolic state and affect the overall physiology. Similarly, changes in skeletal muscle homeostasis can induce system level pathologies such as obesity, diabetes, etc. Disruption or alteration of systemic or muscle tissue metabolism can extend to MuSCs. While the evidence is limited, stressed/dysfunctional MuSCs may potentially impact tissue level or system level metabolic changes.

Physiological regulation of metabolism is bi-directional, in that internal muscle metabolism and systemic metabolic state can impact each other (Figure 1). The systemic metabolic state of the organism can define the composition and metabolic activity of skeletal muscle, and conversely, alteration in skeletal muscle metabolism due to genetic or pathological conditions can induce secondary systemic metabolic imbalances. Moreover, a negative feedback loop can form where de-regulation of systemic metabolism due to altered muscle metabolism can lead to further deterioration in skeletal muscle function.

Muscle fibers can be classified into two broad subtypes: slow and fast (Rolfe and Brown, 1997; Bassel-Duby and Olson, 2006; Schiaffino and Reggiani, 2010; Picard et al., 2011). This classification of myofibers is based on their expression of contractile protein isoforms, preference for energy generation pathway and oxidative capacity, as well as the type of innervation (Figure 1). Slow-twitch fibers preferentially utilize fat as a substrate for energy production, and therefore show higher endurance and oxidative capacity. By contrast, fast-twitch fibers rely on carbohydrates for energy generation, and since glycolysis is less efficient in ATP production, fast-twitch fibers show less oxidative capacity and higher fatigability. Some fast-twitch fibers are oxidative in nature and display intermediate characteristics of slow and fast fibers.

Most muscles contain both fast- and slow-twitch fiber types, but different muscles display distinct proportions of slow and fast myofibers: while some muscles such as the soleus, vastus intermedius, wrist flexors, and extensors are largely composed of slow-twitch fibers, others such as the vastus lateralis, gastrocnemius, and extensor digitorum longus are enriched in fast-twitch fibers. The proportion of slow and fast-twitch fibers in specific muscles may change according to the type of physical activity. Fiber-type enrichment that is regulated by physical activity is most readily observed in athletes—muscles of marathon runners are enriched in slow-twitch fibers that provide continued contractility promoting endurance, whereas sprinters show more fast-twitch fibers providing rapid contraction for bursts of speed (Costill et al., 1976a, b; Harber and Trappe, 2008; Gollnick et al., 2019; Trappe et al., 2019). Oxidative and glycolytic fibers may exhibit metabolism-dependent interconvertibility. Under extended periods of higher energy availability and low body metabolic rate, oxidative fibers can convert into glycolytic fibers (Mootha et al., 2003; Willems et al., 2015). Similarly, under conditions where the body increases metabolic rate to increase endurance capacity, glycolytic fibers convert into oxidative fibers (Fitzsimons et al., 1990). In pathological or stress conditions too, fiber-type switching is observed. For example, muscle from insulin-resistant and diabetic patients contains a greater proportion of fast-twitch fibers, whereas people on endurance or resistance training display more slow-twitch fibers (Lebrasseur et al., 2011; Colbeeg, 2018). Diabetic or obese patients on resistance training show an improvement in insulin resistance and increased basal metabolic rate (Zanuso et al., 2009; Willis et al., 2012).

Diet plays an important role in physiological metabolism. Studies in mice have shown that a high-fat diet regimen causes loss of muscle mass and reduced capacity for muscle regeneration (Woo et al., 2011). A similar observation has been made in obese and diabetic patients. The increase in visceral body fat caused by increased fat intake or non-alcoholic fatty liver disease (NAFLD) triggered by diabetes is linked to negative regulation of muscle mass (Samuel and Shulman, 2012). In a healthy subject, skeletal muscle is responsible for 85% of insulin-dependent glucose absorption (Defronzo et al., 1981a). The development of insulin resistance in muscle leads to increased glucose levels in blood and, in chronic condition, leads to type 2 diabetes. In diabetes, excess circulating glucose is converted to fat, which gets deposited not only in the visceral cavity but also in skeletal muscle, in the inter- and intra-myofiber space (Kelley et al., 1992; Bonadonna et al., 1993). This ectopic fat deposition negatively affects muscle mass and regenerative capacity. Myopathic conditions such as Duchenne muscular dystrophy (DMD) are often associated with obesity and insulin resistance (Rodríguez-cruz et al., 2015). Therefore, imbalances in the level of circulating glucose or fat metabolites directly or indirectly affect skeletal muscle. These studies firmly link systemic metabolism with skeletal muscle tissue metabolism, where a balance between energy intake (diet) and basal metabolic rate defines the health and functionality of skeletal muscle.

### Skeletal Muscle Metabolism and MuSCs

Skeletal muscle regeneration is critically dependent on MuSC function. Under physiological stress, injury, or pathological conditions, the activity of MuSCs determines the extent of regeneration and tissue recovery. MuSCs perdure in adult muscle in a mitotically quiescent or non-dividing state. While it is clear that quiescence is a hypo-metabolic state compared to proliferating or differentiated states, there is increasing evidence that the quiescent state is not a passive one induced by exit from cell cycle, but an actively maintained metabolically adapted state (Ryall et al., 2015b; Pala et al., 2018; Dell’Orso et al., 2019). Thus, although quiescent cells show very low macromolecular synthesis, in that DNA replication is absent and both transcriptional activity and protein synthesis are substantially suppressed, specific metabolic adjustments are made such that the molecular circuitry remains in a poised state to respond quickly and effectively toward cell cycle re-entry and myogenic differentiation. Recent findings suggest that intrinsic programs in dormant MuSC are influenced by extrinsic signals received from the local or systemic environment to affect quiescence and cell fate (Varga et al., 2016; Du et al., 2017; Verma et al., 2018). It stands to reason that circulating metabolites would also impact MuSC function, but direct tests of this link are lacking.

The number of MuSCs associated with each multinucleated fiber depends on the fiber-type (Rats et al., 2001; Christov et al., 2007): slow-twitch fibers contain more MuSCs than fast-twitch fiber (Figure 1). While fiber types clearly differ in their metabolic state, there are confounding factors that make it difficult to conclude that the number of fiber-associated MuSCs is a causal outcome. Fiber-type specification is a composite outcome of multiple factors that include expression of different contractile protein isoforms, frequency and strength of nerve contraction, and the metabolic program (Hughes and Blau, 1992). Interestingly, MuSCs associated with slow fibers have been found to display metabolic and transcriptional differences from fast fiber-associated MuSCs (Motohashi et al., 2018). The slow fiber-associated MuSCs possess greater regeneration and self-renewal capacity, whereas fast-type fiber-derived MuSCs have higher differentiation capacity. Moreover, slow or fast fiber-associated MuSCs are dedicated to regeneration of respective fiber-type only (Motohashi et al., 2018).

Fiber-type switching is influenced by metabolic state as evidenced by alterations in disease conditions such as obesity and diabetes. The exposure of 3-week-old mice to a high-fat diet (60% of caloric intake from fat) for 3 weeks induces obesity and MuSCs show reduced numbers and function, leading to defective muscle regeneration (Woo et al., 2011). The depletion and reduced activity of MuSCs under these conditions might reflect a switch in fiber-type or extrinsic signals. Conversely, a recent study shows that mice fed with a calorie-restricted diet (70% of the normal caloric value) show an increase in MuSC number, increased oxidative capacity of the stem cells due to increased mitochondrial content, and increased proliferative capacity (Cerletti et al., 2012). Similarly, a calorie restriction mimetic drug metformin delays MuSC activation by inhibiting the downstream effector of the mTOR pathway (Pavlidou et al., 2019). MuSCs isolated from calorie-restricted mice also exhibit increased transplantation efficiency (Cerletti et al., 2008), a finding that contradicts an earlier report where native MuSCs containing less mitochondria showed improved transplantation efficiency (Rocheteau et al., 2011). A metabolomic and transcriptomic profiling of MuSCs isolated from metabolically altered mice could provide a better understanding of metabolic differences of these cells from MuSCs isolated from control mice.

The exposure of MuSCs to the systemic circulation is likely to be high, since MuSCs have been shown to be preferentially located in the proximity of capillaries. Muscle types differ in the degree of vascularity: slow muscles are more highly vascularized than fast muscles (Christov et al., 2007). However, whether signals emanating from muscle fiber or from proximal capillaries impact MuSC number is not established. Cross-talk between MuSC and endothelial cells has been recently reported (Verma et al., 2018). Quiescent and activated MuSCs express high levels of VEGFA protein, which is a potent recruitment factor for endothelial cells, and promotes vascularization. Interestingly, MuSCs located closer to capillaries were found to be more quiescent than distant MuSCs, which may be explained by the finding that endothelial cells secrete the Notch ligand Dll4, activating Notch signaling which is required for the maintenance of quiescence. However, oxygenation and cytokine signaling from capillaries or endothelial cells are only one factor, since the observations of Latil et al. (2012) reported the recovery of viable MuSCs from 17-day post-mortem human muscle tissue. These MuSCs not only survived severe hypoxic conditions but showed characteristics similar to deeply quiescent MuSCs with higher mitochondrial content. It seems unlikely that cadaveric endothelial cells would continue to signal to MuSCs to maintain their deep quiescence state. It is possible that MuSCs located distant from capillaries are molecularly different from the population that are located proximal to the capillaries, and may be intrinsically more resistant to hypoxia. MuSCs isolated from 2 days post-mortem mice show reduced dependency on oxidative phosphorylation (OXPHOS) but maintain glycolytic activity (Pala et al., 2018). Unlike MuSCs isolated from 17-day post-mortem humans, MuSCs from 2-day post-mortem mice show reduced mitochondrial content and mitochondrial membrane potential. Further investigation is needed to establish the specific role of vascularization in maintenance of MuSC quiescence and the mechanism of severe hypoxia resistance.

Thus, while MuSCs in their niche are undoubtedly impacted by multiple cell types in their vicinity, and by the metabolic state of the organism, little is known about the potential influence of MuSCs on the tissue or systemic metabolism (Figure 1). It may well be that a small number of hypo-metabolic stem cells are unlikely to influence tissue metabolic state, but in the absence of any information, this conclusion may be premature. It is therefore imperative to study the basal metabolic state of MuSCs, the flux of metabolites between different cellular components of skeletal muscle and their alteration under physiological and pathological conditions.

### Metabolic Signaling in MuSCs and Skeletal Muscle

**FIGURE 2:**
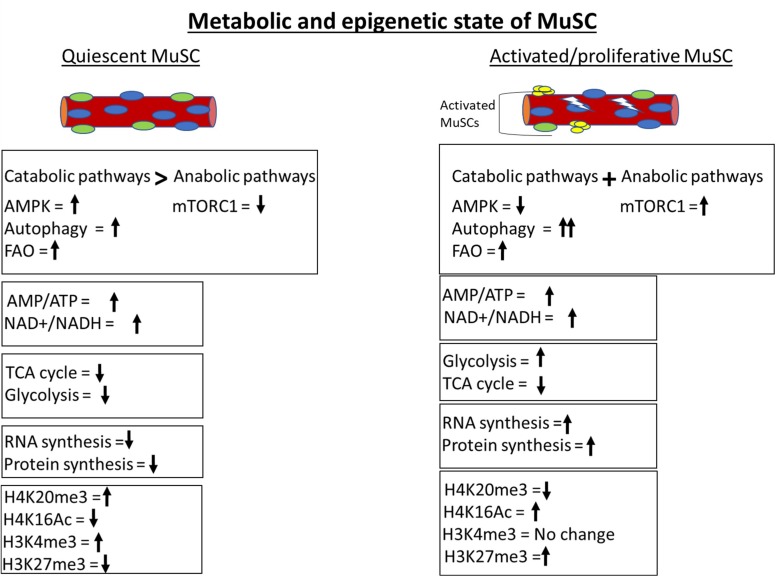
Metabolic and epigenetic state of MuSCs. Quiescent MuSCs are metabolically and transcriptionally less active and conserve ATP by inhibiting anabolic processes. MuSCs fulfill their energy requirement through catabolic processes like autophagy and fatty acid oxidation (FAO). The histone deacetylase SIRT1 is upregulated in quiescent MuSCs and keeps H4 histone in a deacetylated state. The genome of quiescent MuSCs is highly condensed and transcriptionally less active due to H4K20me3-mediated constitutive heterochromatinization. The activation of MuSCs leads to immediate upregulation of glycolytic pathway to fulfill the cellular anabolic requirements. Anabolic processes are more prominent in activated MuSCs compared to catabolic processes as depicted by activation of master anabolic regulator mTORC1 and further increment in autophagy level.

The quiescent state of adult stem cells including MuSCs shares some similarities with hibernation, where cells are maintained at a minimal energy state and required energy is derived from the catabolism of stored macromolecules. Inhibition of anabolism and activation of catabolic processes are an important adaptation to minimize the energy consumption to keep cells in a quiescent state (Fiacco et al., 2016; García-prat et al., 2016a, b; White et al., 2018). For convenience, muscle or MuSC metabolic signaling can be classified into catabolic and anabolic type (Figure 2).

### Catabolic Signaling

Slowing of metabolism leads to reduced ATP generation reflected in higher cellular AMP/ATP or ADP/ATP ratios, which in turn causes activation of AMP-activated kinase (AMPK) (Box 4). AMPK, which maintains the cellular energy state by suppressing anabolic processes and enhancing catabolic processes to generate more ATP, is a central metabolic sensor that requires AMP for its enzyme activity. In the context of muscle, activities such as endurance exercise and resistance training activate adenylate cyclase, which catalyzes hydrolysis of ATP to generate ADP or AMP. This leads to activation of AMPK, which increases transport of glucose and increased FAO to generate more ATP, and inhibits glycogen and protein synthesis to prevent consumption of ATP in these anabolic processes (Kjøbsted et al., 2018). High levels of ATP inhibit activity of AMPK, providing a feedback loop for control of the pathway. AMPK also regulates three other key metabolic regulators: PGC1a, SIRT1, and mTORC1. PGC1a is a master regulator of mitochondrial biogenesis, and AMPK activates PGC-1a through phosphorylation, which in turn induces mitochondrial biogenesis to increase ATP production (Handschin et al., 2007). The activation of histone deacetylase (HDAC) SIRT1 is dependent on NAD+ redox potential and AMPK has been reported to increase NAD+/NADH redox potential in response to muscle contraction (Ryall, 2013). SIRT1 modulates PGC1a activity by deacetylation (Gerhart-hines et al., 2007). mTORC1 is a central activator of anabolic processes especially protein synthesis: under energy-deprived conditions, AMPK inhibits mTORC1 activity and ensures that limited ATP levels are not diverted toward protein synthesis.

**BOX 4.**

AMPK signaling in muscle. AMPK is a molecular sensor whose activation is dependent on AMP/ATP or ADP/ADP ratio. Activated AMPK can activate autophagy through ULK1, p27, or FoxO3. AMPK inhibits mTROC1 through TCS or Raptor protein to minimize the use of ATP in biosynthetic pathways. AMPK also increases mitochondrial biogenesis by activating PGC1α.

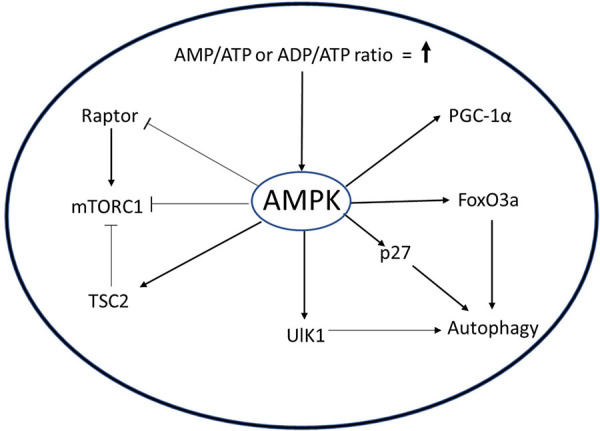

The AMPK pathway plays an important role in the regulation of MuSCs. When activated out of quiescence, MuSC employ autophagy to degrade cellular organelles and proteins to rapidly gain precursors required for the burst of macromolecular synthesis needed to prepare for cell doubling and sustained proliferation (Fiacco et al., 2016). Targeted inhibition of autophagy in quiescent MuSC by genetic deletion of Atg7, a crucial component of autophagosome formation, causes cell death by senescence and mitochondrial dysfunction (García-prat et al., 2016a). AMPK acts as an activator of autophagy either by inducing expression of cell cycle inhibitor p27 and Unc51-like autophagy activating kinase 1 (ULK1), or by inhibition of mTORC1 pathway via phosphorylation of TSC2 and Raptor (Koopman et al., 2014; Rion et al., 2019). White et al. showed that in activated MuSC from young mice, AMPK phosphorylates p27, causing its translocation to the cytoplasm (White et al., 2018). The cytoplasmic p27 activates autophagy in MuSCs, simultaneously suppressing apoptosis and senescence. Interestingly, in MuSCs from aged mice, inactivation of AMPK causes reduced autophagy and increased apoptosis, suggesting that AMPK supports maintenance of quiescence.

Apart from autophagy, AMPK is shown to be associated with other metabolic pathways in MuSCs. Non-canonical Sonic Hedgehog signaling activates AMPK during muscle injury, leading to Warburg-like glycolysis and promoting muscle regeneration (Fu et al., 2015). A similar mechanism is employed by cancer cells for rapid proliferation, where they metabolize glucose under aerobic conditions, generating downstream metabolites that can be drawn off for macromolecular synthesis, and is termed the “Warburg effect” (Warburg, 1956). Surprisingly, knockout of AMPKa1, the dominant isoform of AMPK in MuSCs, also showed an increase in Warburg-like glycolysis in freshly isolated MuSCs and inhibited regeneration by promoting self-renewal of MuSCs (Theret et al., 2017). Possibly, AMPK activity is regulated at a set point and either increase or decrease leads to Warburg-like effect, potentially by different mechanisms. The knockdown of AMPKα1 induces activity of lactate dehydrogenase (LDHA) enzyme, which promotes Warburg-like glycolysis in MuSCs (Theret et al., 2017). Concomitant reduction in the expression of PGC1a and b and reduced activity of citrate synthase suggested a decrease in OXPHOS. Interestingly, *in vivo* electroporation of a LDHA expression construct in muscle increases MuSC self-renewal. Obesity-mediated inhibition of AMPK activity impedes MuSC activation and muscle regeneration (Fu et al., 2016). Moreover, attenuation of regeneration in obese mice could be reversed by pharmacological activation of AMPK using the AMP mimetic, AICAR. Like AICAR, trimetazidine (TMZ) is a metabolic modulator that stimulates expression of myogenic and oxidative genes, thus improving myogenesis in aged muscle (Belli et al., 2019). Both AICAR and TMZ can induce differentiation of MuSC *in vitro*. Chronic activation of AMPK by AICAR leads to a switch from fast to slow fibers and, as mentioned above, differential association of MuSC has been reported with these fiber–types (Ljubicic et al., 2011), suggesting a potential regulation of MuSC numbers via AMPK levels. Thus, while there is clear evidence that AMPK is an important metabolic regulator at the level of both skeletal muscle tissue and MuSCs, the cross-talk between cellular and tissue levels of regulation through the AMPK pathway has not yet been investigated. It would be important to understand how alteration in AMPK levels in MuSC affect muscle tissue, and vice versa.

### Anabolic Signaling

The anabolic signaling reported to play an important role in MuSCs and in skeletal muscle metabolic regulation involves mTORC1, IGF-1, and FoxO family proteins. mTORC1 is a key cellular sensor for nutrients and integrates extrinsic mitogenic signals with internal nutrient levels to regulate the rate of protein synthesis. AMPK and low glycolytic flux inhibit mTORC1 activity in quiescent MuSCs and thus inhibit muscle regeneration (Lee et al., 2009). Most information regarding extracellular signaling and MuSCs in muscle regeneration has been gained from understanding local changes in microenvironment or the niche. A landmark study that implicates a systemic signaling process in regulating MuSC function showed that injury in one hind limb muscle in a mouse leads to molecular changes in MuSCs located in the muscle of the contralateral uninjured hind limb (Rodgers et al., 2014). MuSCs in the uninjured muscle showed signs of early activation including increased cell size, increased numbers of mitochondria, and an accelerated G0–G1 entry. However, as these remotely activated cells do not proliferate (i.e., do not proceed to S phase) in response to distant injury cues, the authors coined the term “G_alert_” quiescent cells to reflect the fact that these cells return to quiescence after a transient activation, similar to the competence phase of growth factor signaling defined in the classical competence-progression model (Pledger et al., 1978). Importantly, G_alert_ MuSCs showed hepatocyte growth factor (HGF)-dependent upregulation of mTORC1 function. HGF is the earliest signaling cue known to activate MuSC in response to injury (Miller et al., 2000). Activation of the mTOR pathway by MuSC-specific genetic ablation of mTOR inhibitor (TSC1) induces a “G_alert_” phenotype, while conversely, inhibition of mTOR pathway by ablation of Raptor prevents MuSCs from exiting quiescence, pointing to the essential role of the mTOR pathway in MuSC activation.

Similar to mTOR, several other anabolic pathways are implicated in MuSC activation and proliferation. IGF-1 is an important regulator of the myogenic program: low levels of IGF-1 are associated with muscle loss (sarcopenia), while ectopic expression of IGF-1 causes reversal of the sarcopenic phenotype (Musarò et al., 2001). IGF-1 is a known activator of protein synthesis and induction of IGF-1 in muscle causes increased amino acid transport and inhibition of protein degradation (Clemmons, 2013). Knockout of the IGF-1 receptor impairs satellite cell proliferation and differentiation (Zhang et al., 2010). Similarly, FoxO transcription factors are involved in the regulation of cellular metabolism, proliferation, and oxidative stress resistance (Gross et al., 2008). In muscle, FoxO proteins are central to muscle atrophy that results from disuse (Mammucari et al., 2007). They induce the atrophy-related ubiquitin ligase atrogin-1 to enhance protein catabolism and hence muscle atrophy. FoxO3 independently regulates both autophagic/lysosomal and ubiquitin-mediated protein degradation pathways. Increased muscle protein catabolism by FoxO3 produces more amino acids to be used under starvation conditions. Transcriptional activity of FoxO1 and FoxO3 are required for the regulation of glucose uptake and inflammation in muscle (Lundell et al., 2019). FoxO3 transcription factors not only regulate muscle metabolism, but also induce expression of Notch1 and Notch3 to promote quiescence during MuSC self-renewal (Gopinath et al., 2014). By contrast, during MuSC activation, FoxO3 in association with Pax7 recruits RNA polymerase II and the transcription initiation complex to MyoD promoter (Hu et al., 2008). FoxO3 also interacts with MyoD to orchestrate super-enhancer hotspot assembly during the early phase of muscle differentiation (Peng et al., 2017). Thus, the evidence suggests that FoxO transcription factors might be important metabolic integrators in MuSCs and warrant further investigation.

In summary, quiescent MuSCs display low metabolic activity and show active inhibition of anabolic pathways to conserve energy, along with induction of catabolic pathways that prevent senescence by regulation of proteostasis. Pathological conditions like age-related sarcopenia are associated with a progressive decline in MuSC catabolic activity. Inhibition of autophagy in young mouse MuSC recapitulates characteristics of MuSC from aged mice. By contrast, activated or proliferative myoblasts mainly rely on flux between anabolic pathways in conjunction with catabolic pathways to synthesize macromolecules for rapid proliferation. Together, these findings further strengthen the idea that the metabolic state of MuSC is actively maintained, and perturbation of MuSC-intrinsic metabolic pathways can abrogate MuSC activity.

## Metabolic Signature of MuScs

For the past decade, the primary focus of research on MuSC regulation has been on identifying epigenetic, transcriptional, and signaling signatures that illuminate the control of entry and exit from quiescence, and their ability to serve as regenerative units. While metabolic implications of transcriptional signatures have been imputed from the functions of individual genes or gene networks, few studies have directly addressed the metabolic state of MuSCs (Figure 2). However, studies on induced quiescence in a variety of cultured cells and metabolic analysis of other stem cells provide some clues (Lemons et al., 2010; Takubo et al., 2013; Schell et al., 2017; Wang et al., 2018; Li et al., 2019). The available evidence suggests that like transcription and translation, the metabolic state of MuSCs is also actively maintained via intrinsic and extrinsic control.

Terminally differentiated myofibers possess an abundance of mitochondria and utilize OXPHOS as a major source of energy (Leary et al., 1998; Kraft et al., 2006). By contrast, activated MuSCs and their proliferating progeny myoblasts are heavily dependent on glycolysis not only for ATP production but also for the generation of glycolytic intermediates for the synthesis of complex biomolecules (Pala et al., 2018). Fetal myogenic cells also show higher glycolysis flux to support the increased demand for macromolecule biosynthesis. The rate of glycolysis in MuSCs is reported to increase as soon as they break quiescence, reaching threefold within 3 h of activation. Interestingly, activation of MuSCs also leads to rapid mitochondrial biogenesis (Ryall et al., 2015b). However, despite an increase in the number of mitochondria, there is no drastic change in OXPHOS activity even after 20 h of activation, supporting the preference for glycolysis for rapid energy generation and macromolecular synthesis.

The metabolic state of activated MuSC is under transcriptional control of the Yin Yang 1 (YY1) transcription factor (Chen et al., 2019). YY1 not only binds and suppresses the expression of TCA cycle genes, but also stabilizes the glycolytic gene regulator HIF1a during activation. HIF1a increases the glycolytic rate by inducing the expression of glycolysis genes. In contrast to the finding with YY1, other reports suggest increased expression of TCA cycle genes during MuSC activation (Pallafacchina et al., 2010; Pala et al., 2018; Dell’Orso et al., 2019). Though TCA cycle gene expression was enhanced, the TCA cycle activity remained lower compared to glycolysis during MuSC activation. Possibly, the selective inhibition of TCA cycle genes by YY1 could be the reason of TCA cycle inhibition during MuSC activation. For aerobic glycolysis, continued availability of oxygen and glucose is critical, and likely to be provided by the highly oxygenated location of MuSCs in proximity to capillaries. Despite having similar mitochondrial content, MuSCs isolated from aged mice show an increased dependence on glycolysis (Pala et al., 2018). Gene expression analysis of old MuSCs reveals decreased expression of genes involved in TCA cycle, OXPHOS, and fatty acid metabolism in comparison to young MuSCs. Given the low metabolic state of quiescent MuSCs, it is likely that age-related systemic metabolic changes and alteration in local niche induce these metabolic shifts. However, there is as yet, no explicit analysis of these linkages.

Genome-wide expression studies show an enrichment of glycolysis genes in proliferating myoblasts, corroborating the biochemical data (Pallafacchina et al., 2010; Liu et al., 2013; Ryall et al., 2015b; Pala et al., 2018; Dell’Orso et al., 2019). Quiescent MuSCs, by contrast, show an enrichment of genes controlling fatty acid oxidation (FAO), as seen in other quiescent stem cells such as hematopoietic stem cells (HSCs) and neural stem/progenitor cells (NSPCs) (Ito et al., 2012; Zamboni et al., 2017). Elevated levels of pyruvate kinase M1/2 (PKM2) and pyruvate dehydrogenase kinase 1–4 (PDK1–4) in quiescent HSC suggest a reliance on anaerobic glycolysis (Takubo et al., 2013). PDK inhibits the pyruvate dehydrogenase complex, thus restricting synthesis of acetyl coenzyme A (acetyl-CoA) from pyruvate. The elevated pyruvate is converted into lactate and secreted by the cell. In this scenario, the FAO pathway is an alternate source of acetyl-CoA for HSCs. In the absence of glucose, acetyl-CoA does not participate in histone modifications but gets utilized in the TCA cycle and electron transport chain (ETC) for energy production (Wellen et al., 2009). It has been observed that glucose utilization increases in proliferating MuSCs, but instead of participating in mitochondrial respiration, it mainly serves as a source of nuclear acetyl-CoA in proliferating MuSCs (Yucel et al., 2019). Mitochondrial pyruvate dehydrogenase enzyme (PDH), which converts pyruvate to acetyl-CoA, acts as a controller of glucose-derived acetyl-CoA generation. Constitutive activation of PDH activity by knockdown of PDK enzyme increases overall protein acetylation level, promotes MuSC self-renewal, and inhibits differentiation after muscle injury. Apart from glucose, acetyl-CoA can be generated from fatty acid and protein metabolism. A recent study showed that fatty acid oxidation in peroxisomes, but not in mitochondria, is necessary in the early phase of muscle differentiation (Pala et al., 2018). Inhibition of peroxisomal FAO by chemical inhibitors causes induction of Pax7 and Myogenin expression and leads to precocious differentiation. Freshly isolated proliferative MuSCs show a higher rate of FAO than fetal and perinatal myogenic cells, suggesting an important role of FAO in adult MuSCs, potentially for regeneration.

Thus, there is a clear paradigm for the active mechanisms by which MuSCs keep their transcriptional and metabolic activity in check during quiescence. The identity of regulators that define the basal metabolic state of MuSCs is as yet unknown and represents an important area of research. Such molecules are likely to play a central role in regulation of quiescence in MuSCs, given the direct role of metabolites in enzymes that impose epigenetic regulation. Further, the mechanisms by which these intrinsic programs within MuSC might be regulated by signaling from the niche and in response to the systemic metabolic state would be a key step forward in understanding the interconnections between overall physiology and specific stem cell populations.

## Metabolic–Epigenetic Axis of MuSc Regulation

Several metabolites are directly involved in the regulation of epigenetic modification, whereas many others participate indirectly (Box 3). A detailed description of all these metabolites and their function is beyond the scope of this review: excellent coverage of this topic is provided by Etchegaray and Mostoslavsky (2016), Gao et al. (2018), Kulkarni et al. (2019), and Reid et al. (2018). Briefly, metabolites such as acetyl-CoA and S-adenosylmethionine (SAM) act as direct substrates for histone modification, whereas α-ketoglutarate (αKG), NAD+, and FAD act as co-factors for chromatin-modifying enzymes. Acetyl-CoA is an essential substrate for histone acetyltransferase enzymes (HATs) for histone acetylation and SAM is the methyl group donor for histone methyltransferase (HMT)-mediated modifications. αKG and FAD serve as co-factors for histone demethylases such as TET-family demethylases, JmjC family histone methylases, and LSD family histone demethylases. NAD+ is required for HDAC activity. Despite a great deal of information showing that regulation of epigenetics through metabolism is critical, their connection and importance in stem cell regulation have been realized very recently.

The first explicit analysis of metabolic contributions to epigenetic regulation in quiescent MuSCs came from studies of Ryall et al. who reported the role of sirtuins, HDACs that are associated with repression of gene expression (Ryall et al., 2015b). Expression of SIRT1 is enriched in quiescent MuSCs. SIRT1 uses NAD+ as a cofactor for its enzymatic function and, therefore, works as a redox sensor. Quiescent MuSCs exhibit a higher NAD+/NADH ratio, leading to decreased acetylation at H4K16 (the histone modification regulated by SIRT1). Upon activation of MuSC, glycolysis is stimulated and the NAD+/NADH redox level decreases, leading to deactivation of SIRT1 and concomitant increases in H4K16 acetylation at myogenic loci and their subsequent activation. These results are supported by transcriptomic studies, where an enrichment of FAO genes was found in quiescent MuSCs and upregulation of glycolytic genes was observed in activated MuSCs (Pallafacchina et al., 2010; Liu et al., 2013) (Figure 2). Therefore, the redox level controls molecular regulators like sirtuins according to the metabolic requirement of cellular state, but the identity of central pathways/genes that dictate redox level in MuSCs is still elusive.

Acetyl-CoA is a common metabolite linking glycolysis, FAO, and protein metabolism pathways. The cytoplasmic ATP citrate lyase (ACL) converts citrate into acetyl-CoA, which is utilized in fatty acid synthesis. Interestingly, nuclear ACL can catalyze the same reaction in nucleus, but here, acetyl-CoA is utilized by HAT to acetylate specific histone proteins (Das et al., 2017). A recent study shows that the level of histone acetylation is higher in proliferating MuSC compared to quiescent and differentiating muscle cells (Yucel et al., 2019). The genes affected by increased acetylation level are related to self-renewal and cell cycle regulation. Higher acetylation inhibits muscle differentiation, and promotes proliferation and self-renewal of MuSCs. Reducing the level of histone acetylation by knocking down ACL in MuSC shows a generalized decrease in histone H3 acetylation and induction of differentiation (Das et al., 2017). By contrast, induction of PDH activity by knocking down PDK enzyme to induce acetyl-CoA generation causes increased muscle differentiation. Similarly, overexpression of ACL increases acetyl-CoA levels in MuSC and promotes myogenesis by acetylation of MyoD regulatory regions. ACL activity is regulated by the IGF-1/PI3K/AKT pathway (Das et al., 2015), showing that extrinsic signaling can directly impact MuSC function through altered intracellular metabolite levels. Thus, the level of acetylation appears to define the proliferative, self-renewing, or differentiating characteristics of MuSCs.

In homeostatic conditions, the quiescent MuSC genome, while substantially less transcriptionally active than in proliferating MuSC, is enriched with “activating” H3K4me3 marks. Upon activation, H3K4me3 marks are not lost but repressive H3K27me3 marks increase (Figure 2). The deposition of H3K27me3 is regulated by the histone methyl transferase Ezh2, a component of the conserved polycomb repressive complex 2 (PRC2), and Ezh2 expression is upregulated in activated MuSCs. The methyl donor SAM is utilized by both histone and DNA methyl transferases. SAM is generated through the one-carbon metabolic cycle, where serine is the predominant source of carbon. Interestingly, several enzymes of the serine biosynthetic pathway are upregulated in activated MuSCs (Ryall et al., 2015a). Possibly, increased levels of Ezh2 enzyme and SAM substrate are responsible for increased H3K27 methylation in activated MuSC.

Histone modifications also appear to limit the expression of myogenic genes via regulation of chromatin compaction. The histone methyltransferase Suv4-20h1 is expressed specifically in quiescent MuSCs and drastically suppressed after MuSC activation (Boonsanay et al., 2016) (Figure 2). Suv4-20h1 enzyme deposits H4K20me2 epigenetic mark and induces facultative heterochromatization of chromatin. H4K20me2 also acts as a substrate for H4K20me3 modification, which marks constitutive heterochromatin. The level of H4K20me3 methylation mark reduces drastically during MuSC proliferation. Transient silencing or knockout of Suv4-20h1 in MuSCs causes decreased chromatin compaction, aberrant activation of MyoD expression, and defective muscle regeneration. These findings suggest that Suv4-20h1-mediated heterochromatin formation is vital for maintenance of MuSC quiescence and regulation of gene expression. The Suv4-20h1 histone lysine methyltransferase (KMT) enzyme utilizes SAM as a substrate for histone methylation. However, the impact of changes in SAM levels on global as well as enzyme-specific histone methylation status is undocumented and warrants further analysis.

Together, the evidence suggests that the epigenetic state of MuSC is actively maintained and that external signals impact the cellular state by altering metabolite levels. Further work is needed to establish the role of specific metabolites in the transitions between quiescence, proliferation, and differentiation during development and regeneration.

## Summary and Open Questions

The study of metabolic regulation of and by stem cells has gained importance, especially in the context of whole organismal physiology. As with the epigenome, transcriptome, and proteome, it is becoming increasingly clear that the metabolome is critical to cellular identity and cell fate. Three major factors define the systemic metabolic state of an organism: (1) caloric value of the food consumed, (2) basal metabolic rate of the body coupled to physical activity, and (3) pathological condition(s). The systemic metabolic state regulates the organ and tissue level metabolism. Currently, the quest is to understand the role of metabolism in development, disease, and regeneration.

In this review, we have attempted to connect the dots between cellular, tissue, and system level metabolism by focusing on skeletal muscle tissue and its resident adult MuSC. Through intrinsic and extrinsic factors, the cellular metabolism of MuSC is maintained such that it supports the quiescent state. It is characterized by induction of catabolic activity through activation of autophagy and low anabolic rate by the suppression of glycolysis and oxidative metabolism. Upon activation, MuSCs need material and energy to rapidly synthesize proteins and membranes, which is fulfilled by the activation of glycolysis. Eventually, the heightened metabolic activity supports new transcription and DNA synthesis, leading to proliferative expansion. By contrast, myofibers utilize OXPHOS to meet the high-energy demands of terminally differentiated tissue whose primary function is the ATP-consuming task of nerve-dependent contraction. Skeletal muscle fibers along with other less abundant cell types in muscle such as endothelial cells, resident macrophages, and fibro-adipogenic precursors combine to create the physical, chemical, and temporal niche of MuSC and directly influence their state of quiescence/activation.

Several studies have shown the impact of muscle tissue metabolism on the activity of MuSCs, but under normal conditions, the converse, i.e., influence of MuSC metabolic state on skeletal muscle metabolism or systemic metabolism, may not be substantial, due to their small number and very low rate of turnover. However, under regenerative or pathological conditions, the influence of MuSCs cannot be ignored. Alterations in extrinsic signaling, not necessarily under pathological situations, are capable of affecting MuSCs chronically. Ultimately, these changes in MuSC may hamper the regeneration of skeletal muscle tissue. However, to date, there are no studies that explicitly investigate the impact of altered metabolic state of MuSCs on skeletal muscle metabolic state under normal or pathological conditions. It is likely that with the advent of single cell technologies, the increased sensitivity and ability to sample heterogeneity of responses in a given condition will yield new information on this important question.

Metabolism and epigenetic regulation are closely linked processes. Extrinsic signals, intrinsic regulators, activity of metabolic enzymes, and pathological conditions define the impact of cellular metabolic state on gene regulation. Several metabolites participate directly in epigenetic modification, whereas many others do so indirectly. Very little information is available about metabolic regulation of MuSCs and its impact on MuSC epigenetic state. Relatively more studies have linked systemic metabolism with skeletal muscle metabolism, as their physiological interconnectedness is readily accessible, which is reflected and measurable in disease.

Despite extensive research over the past several decades, several questions remain unanswered: (1) How do myopathies impact MuSC-intrinsic and systemic metabolism? (2) How does alteration in diet affect muscle metabolism and MuSC metabolism? (3) During the course of development, how does the systemic metabolic state affect skeletal muscle development, and attainment of MuSC quiescence? (4) What are the metabolic changes associated with MuSC in aging and muscle wasting? (5) How are the changes in metabolic state during MuSC activation, proliferation, regeneration, and differentiation linked to epigenetic state and gene expression? (6) Are there molecular mediators that permit interlinking of MuSC, muscle tissue, and systemic metabolism, and what is their nature? The long-term hope of understanding these connections is that knowledge about well defined metabolic pathways, their regulatory steps, and availability of a variety of small molecules to intervene in these pathways may offer new therapeutic avenues, or subdue secondary pathological symptoms in myopathies by simple metabolic interventions.

## Author Contributions

Both authors contributed to the research, writing, and editing of the manuscript.

## Conflict of Interest

The authors declare that the research was conducted in the absence of any commercial or financial relationships that could be construed as a potential conflict of interest.
